# Use of Artificial Intelligence and Machine Learning for Discovery of Drugs for Neglected Tropical Diseases

**DOI:** 10.3389/fchem.2021.614073

**Published:** 2021-03-15

**Authors:** David A. Winkler

**Affiliations:** ^1^ Monash Institute of Pharmaceutical Sciences, Monash University, Parkville, VIC, Australia; ^2^ Latrobe Institute for Molecular Science, La Trobe University, Bundoora, VIC, Australia; ^3^ School of Pharmacy, University of Nottingham, Nottingham, United Kingdom; ^4^ CSIRO Data61, Pullenvale, QLD, Australia

**Keywords:** machine learning, artificial intelligence, drug discovery, neglected tropical diseases, structure-property relationships

## Abstract

Neglected tropical diseases continue to create high levels of morbidity and mortality in a sizeable fraction of the world’s population, despite ongoing research into new treatments. Some of the most important technological developments that have accelerated drug discovery for diseases of affluent countries have not flowed down to neglected tropical disease drug discovery. Pharmaceutical development business models, cost of developing new drug treatments and subsequent costs to patients, and accessibility of technologies to scientists in most of the affected countries are some of the reasons for this low uptake and slow development relative to that for common diseases in developed countries. Computational methods are starting to make significant inroads into discovery of drugs for neglected tropical diseases due to the increasing availability of large databases that can be used to train ML models, increasing accuracy of these methods, lower entry barrier for researchers, and widespread availability of public domain machine learning codes. Here, the application of artificial intelligence, largely the subset called machine learning, to modelling and prediction of biological activities and discovery of new drugs for neglected tropical diseases is summarized. The pathways for the development of machine learning methods in the short to medium term and the use of other artificial intelligence methods for drug discovery is discussed. The current roadblocks to, and likely impacts of, synergistic new technological developments on the use of ML methods for neglected tropical disease drug discovery in the future are also discussed.

## Introduction

Infectious diseases are responsible for the majority of mortality, morbidity, and loss of productive years of life globally. Although most tropical diseases have some type of chemotherapeutic option available, the cost and relative lack of efficacy, coupled often with rapid development of drug resistance, have resulted in unsatisfactory progress in prevention and treatment of these ailments. For the purposes of this review, neglected tropical diseases (NTDs) are listed in Table 1. Several tropical diseases are among the world’s biggest killers. Table 2 lists the global disease burden caused by the top five diseases. In addition, amebiasis is endemic in many countries and has been estimated to kill 55,000 people each year, making it one of the top tropical diseases in terms of mortality (Shirley et al., 2018).

**TABLE 1:** Tropical diseases included in literature searches and reviewed in this report.

Malaria	Amebiasis	Balantidiasis	Chagas	Giardiasis
Trypanosomiasis	Leishmaniasis	Helminth	Taeniasis	Cysticercosis
Dracunculiasis	Echinococcosis	Trematodiases	Loiasis	Filariasis
Onchocerciasis	Schistosomiasis	Helminthiases	Ascariasis	Hookworm
Trichuriasis	Strongyloidiasis	Toxocariasis	Dengue	Japanese encephalitis
Yellow fever	Arboviral infections	Rabies	Rift Valley fever	Viral hemorrhagic fever
Bartonella	Tuberculosis	Ebola	Buruli Ulcer	Cholera
Shigella	Leprosy	Leptospirosis	Relapsing fever	Trachoma
Treponematoses	Bejel	Pinta	Syphilis	Yaws
Eumycetoma	Paracoccidioido-mycosis	Ectoparasitic infections	Scabies	Myiasis

**TABLE 2:** Global burden of disease due to major tropical infectious diseases (Njogu et al., 2016).

Infection	Global prevalence (millions)	Population at risk (millions)	Annual mortality (thousands)	Disability-adjusted life years (millions)	Regions of highest prevalence
Malaria	198	3,200	584	46.5	Sub-Saharan Africa, Asia, South and Latin America, Middle East, and Pacific Islands
tuberculosis	11	2000	1,100	34.7	Sub-Saharan Africa and Southeast Asia
Leishmaniasis	12	350	51	2.1	India, South Asia, Sub-Saharan Africa, Latin America, Caribbean, and Mediterranean region
Human African trypanosomiasis	0.3	60	48	1.5	Sub-Saharan Africa
Chagas’ disease	10	120	15	0.7	Latin American and Caribbean

The current business model for new drug development favors developed countries with populations or governments able to pay for drugs, allowing the very high development costs (median 2020 cost estimated to be US$1-1.3Bn) to be recouped by companies (Wouters et al., 2020). This is one of the major reasons why most tropical diseases are “neglected.” Given the immense burden of disease, there is clearly an urgent need to develop better treatments for NTDs. One way to achieve this is through the intervention of charitable government funds like the US NIH and DoD, European Union, Medicines for Malaria venture, Bill and Melinda Gates Foundation, and the Wellcome Trust. One aim of the London Declaration of Neglected Tropical Diseases (Molyneux, 2017), inspired by the World Health Organization 2020 roadmap to eradicate or prevent transmission for neglected tropical diseases (https://unitingtocombatntds.org/resources/who-roadmap-ntds/), is to “advance research and development through partnerships and provision of funding to find treatments and interventions for NTDs. Global warming that increases the range of some NTDs, and the pandemic risk that infectious diseases originating from tropical regions pose, is driving renewed interest and urgency on disease preparedness that includes preemptive development of drugs. These programs and factors should drive increased interest and investment in discovery of drugs for NTDs in the future.

NTD drug research is progressing quite strongly using traditional medicinal chemistry discovery methods (see a very recent thematic issue (Ali et al., 2020)). Recent paradigm shifting developments in science and technology promise to improve the efficiency of drug discovery for NTDs. These technologies include robotics and automation that make faster and cheaper synthesis and drug assays possible. These technologies are capable of generating very large and rich data sets that can be used to train machine learning (ML) models or being exploited by other AI-based methods of drug discovery (Ferreira and Andricopulo, 2019). It is very clear that AI and ML methods are creating potentially disruptive paradigm shifts in many areas of science, technology, and medicine.

Recent developments in deep learning have provided powerful new tools for screening large libraries of candidate molecules for promising leads and for rational design of new therapies for many diseases. The application of ML and AI methods to drug and materials discovery and optimization are reviewed in several recent papers (Le et al., 2012; Le and Winkler, 2015). Deep learning methods have made a massive impact in science and technology generally and drug discovery in particular, and recent reviews summarize the state of the art and applications (Ferreira and Andricopulo, 2019; Lavecchia, 2019; Rifaioglu et al., 2019). These computational methods are very fast, accessible to scientists in developing countries, and ideal for screening very large libraries of accessible compounds against specific molecular targets or diseases or for repurposing existing drugs, clinical trials candidates, and approved natural products. Repurposing is very useful because any leads that are discovered have already had their safety in man assessed, so they can be trialed in humans more quickly and cheaply than completely new drugs. The current state of the art in drug repurposing for NTDs was reviewed recently by Klug et al. (2016) and Swinney and Pollastri (Swinney and Pollastri, 2019).

Here the author reviews the application of AI and ML to discovery of drugs to treat NTDs. He focuses exclusively on these computational approaches. Readers interested in structure-based or pharmacophore methods of drug discovery are referred to a comprehensive review of the application of these approaches to malaria, tuberculosis, trypanosomiasis, and leishmaniasis by Njogu et al. (2016). The literature review for the current paper involved searches for relevant papers in Web of Science (WoS) and Google Scholar (for the very recent papers) using search terms for artificial intelligence (AI) and machine learning (ML) and all of the NTDs listed in Table 1. The searches yielded 475 relevant papers. The application of statistical modelling methods like quantitative structure-activity relationships (QSAR) and ML to NTDs is relatively recent, with the first reports of the use of neural networks in 1995 (Almeida et al., 1995). The vast majority of ML research for NTDs has appeared only in the last 20 years and the literature base is still relatively small.

### What Types of AI and ML Methods are Used in Drug Discovery for NTDs?

AI and ML methods are deployed primarily for ligand-based design or discovery of new therapeutics. Unlike structure-based methods that use experimental structures of target proteins for drug design, ligand-based methods look for patterns in sets of small drug-like molecules that describe how their molecular properties modulate their biological activities. These patterns are described mathematically by the well-established and validated QSAR method. QSAR relies on the observation that similar molecules often exhibit similar biological activities and that changes to chemical structure in a series of drug candidates can be correlated with their biological effects. These mappings between structure and biology can provide both qualitative and quantitative predictions of likely drug potency and may also elucidate mechanisms of action at the molecular level. While the latter was the initial use of QSAR methods, greatly expanded data sets and computational power have seen a shift to using QSAR models to predict the biological and physicochemical properties of extremely diverse chemical libraries where there is no longer a common chemical scaffold. These two important purposes of QSAR have been described in detail in a recent paper by one of the “fathers” of the QSAR method, Toshio Fujita (Fujita and Winkler, 2016).

While QSAR was initially a statistical modelling method using linear regression (MLR) and linear logistic regression (LLR), the past three decades have seen greatly expanded application of diverse machine learning methods, principally Gaussian processes (GP), artificial neural networks (ANN) and their Bayesian version (BRANN), support vector machines (SVM, including the SMO implementation) and their Bayesian variant relevance vector machines (RVM), decision trees (DT, including the J48 implementation), random forests (RF) and their variants such as extreme gradient boost (XGBoost), Naïve Bayes (NB), and k-nearest neighbor (kNN) clustering methods. More recently, deep learning (DL) algorithms like deep neural networks (DNN), convolutional neural networks (CNN), generalized adversarial networks (GAN), associative neural networks (AsNN), encoder-decoder networks, and recurrent neural networks (RNN) have exhibited very interesting properties useful for modelling structure-activity relationships. The theory behind these methods is complex and outside the scope of the review. Interested readers are referred to recent papers and textbooks that describe these methods and their applications to drug discovery (Ballester, 2019; Cartwright, 2020).

ML methods map mathematical descriptions of structural and physicochemical properties of small molecules (molecular descriptors) to their biological or physicochemical properties of interest. The most important determinants of ML model quality and predictivity are the size, quality, and diversity of the training data and the quality and relevance of descriptors. The ML algorithms used to generate the models have a much smaller impact on model quality and predictivity (see examples below). One of the main reasons for the current intense interest in deep learning methods is because they offer a potential solution to an important, long-standing problem in the QSAR field, how to objectively generate new, efficient, interpretable molecular descriptors for training models (Winkler and Le, 2017). Given the advantages these ML modelling methods provide, they are being increasingly used to design, discover, and optimize drugs for NTDs (Scotti et al., 2015). In particular, the application of these computational techniques to discover drugs for malaria, tuberculosis, trypanosomiasis, and leishmaniasis has been reviewed by Njogu et al. (2016), for leishmaniasis and trypanosomiasis by Halder et al. (2020), and for tuberculosis by van Wijk et al. (2020)

### Case Studies Using AI and ML to Discover New Drugs for NTDs

Although Table 1 lists a large number of NTDs, our literature review identified papers for only a relatively small subset of these, primarily those with the largest disease burden. These are trypanosomiasis; leishmaniasis; malaria; tuberculosis; plague; and HIV (although an argument could be made that this is neither neglected nor purely tropical). The examples reviewed here are not exhaustive but provide a balanced overview of the methods used, the outcomes, and the NTDs to which they are applied. It is notable that research in this field is dominated by Ekins and coworkers; many of these publications are cited in this review. These researchers used both Bayesian and traditional ML methods to model datasets of compounds active against NTDs and most them are only discussed in *Bayesian Models, Clustering and Visualization* to eliminate redundant discussion.

### Traditional ML Models

Almost all studies using ML to discover drug candidates for NTDs have been published within the past two decades. The use of quantitative structure-activity relationship (QSAR) methods, largely statistical models, for drugs against leishmaniasis and trypanosomiasis prior to 2010 has been reviewed (Castillo-Garit et al., 2012). An overview of recent structure-based, ligand-based, and bioinformatics research aiming at identifying novel inhibitors and promising drug targets for *Mycobacterium tuberculosis* was reported by Alladi (2018). Computational modelling, simulation, and prediction methods are becoming more powerful and accurate, and their accessibility is excellent, a particularly valuable attribute for researchers from developing countries.

#### Trypanosomiasis

One of the first uses of ML methods for NTDs was to solve a formulation problem with benznidazole, a drug used to treat Chagas’ disease (American trypanosomiasis) (Leonardi et al., 2009). Its low water solubility was the rate-limiting step in oral absorption, so chitosan microparticles were used to improve its pharmacokinetic properties. The influence of process parameters such as encapsulation efficiency, size, yield, and dissolution rate is modelled using ANNs, ultimately allowing optimization of oral absorption.

Guerra et al. reported a study in which 72 compounds assayed *in vitro* against the epimastigote form of the Tulahuen 2 strain of *Trypanosoma cruzi* were modelled using ML, with descriptors generated by the CODES software (Guerra et al., 2013). CODES generates topological (molecular graph) descriptors that describe the connectivity and properties of atoms in the training set molecules. They employed dimensional reduction to ensure the number of descriptors was substantially less than the number of training example to avoid overfitting the model. They trained a three-layer neural network with between three and five neurons in the hidden layer to form the model. Forty-two compounds were used as the training set and the remainder formed the test set used to assess the predictive power of the model. The model was moderately successful at predicting the activity of compounds in the test set, with standard errors (SE) of prediction and root-mean-square error (RMSE) values around 0.17 and area under the receiver operator curve (AROC) values of 0.7 (a value of 0.5 is random).

Useful antitrypanosomal drug models have been generated using RF, stochastic gradient boosting (SGB), multivariate adaptive regression splines (MARS), and Gaussian process (GP) regression (Kryshchyshyn et al., 2018). An in-house library of 206 thiazolyl hydrazones, thiopyranothiazoles, isothiocoumarin-3-carboxylic acids, and imidazothiadiazoles with confirmed activity against *T. brucei* was used to train the models. The RF model had the highest predictive power, with SGB and GP being substantially worse and MARS providing poor predictions. The models were trained on the log of the percentage growth, converting percentage growth to approximate EC_50_ values. However, using a logit transform may have improved the predictive power of the models.

**FIGURE 1:**
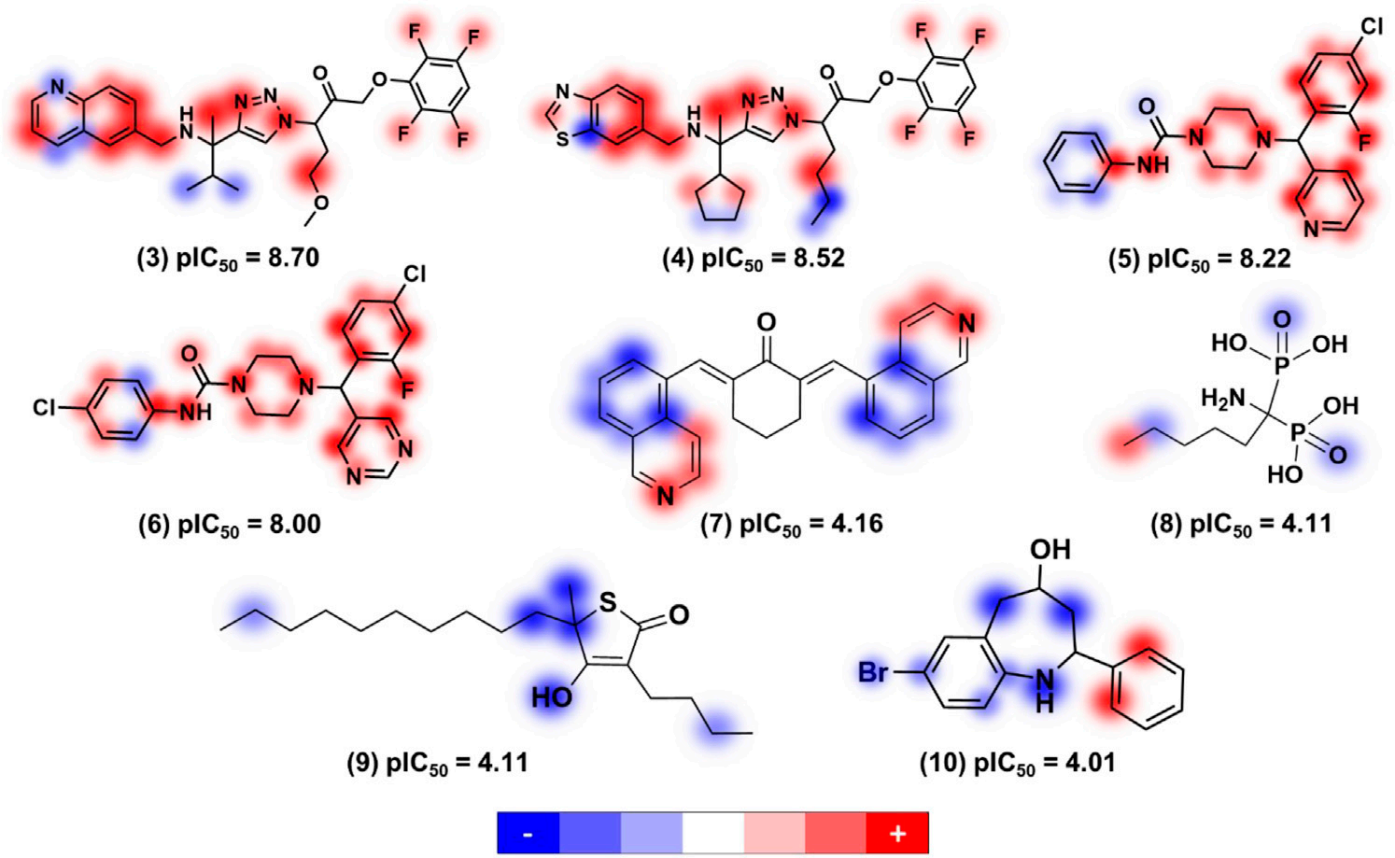
Relevant structural features for trypanocidal activity depicted as contribution maps based on the best kernel based PLS model. Positive, neutral, and negative contributions are depicted in red, white, and blue, respectively, and the color intensity denotes magnitude (de Souza et al., 2019). Creative Commons Attribution (CC BY) license (http://creativecommons.org/licenses/by/4.0/).

The application of ANNs and kernel-based PLS (KPLS) to model 363 compounds with anti-*T. cruzi* activity was reported by de Souza and colleagues (de Souza et al., 2019). Here, the input data was mapped into high-dimensional feature space by nonlinear (kernel) function and linear PLS is carried out in this high-dimensional space (Wang et al., 2015). The models exhibited good predictive ability for the test set compounds, yielding *r* ^2^ and RMSE values of 0.85 and 0.75 for the ANN model, respectively. The KPLS model was used to provide a comprehensive analysis of molecular features improving or degrading the antitrypanocidal activities of molecules (Figure 1).

Luchi and colleagues adopted a different type of molecular descriptor to model the activity of compounds against *T. cruzi* (Luchi et al., 2019). They used charge density topological analysis of molecules bound to the parasite major cysteine protease, cruzain, to code relevant molecular interactions. They also employed feature selection to avoid overfitting the small data set of 17 compounds. SVM was used to generate a binary classification model, as their main aim was to understand the molecular basis of activity rather than predicting activity of new compounds. Although the classification accuracy of the model was essentially the same when using 20 – 87 features in the model, the authors unfortunately chose 87 features instead of the most parsimonious 20 features to generate the models. Given the general tendency of SVM to overfit data (Burden and Winkler, 2015) and the small data set size, use of a larger number of features is not justified. Nonetheless they provided a detailed analysis of the charge features in the active site that modulated activity of cruzain inhibitors.

#### Leishmaniasis

Castillo-Garit et al. reported a study that used traditional ML methods to identify new potential antileishmanial drug leads. A data set of 116 compounds assayed against promastigotes of *Leishmania amazonensis* was used to train kNN, RT, SVM, and ANN models. All ML models provided accuracies between 82 and 91%, for the training set and external test set. A subsequent virtual screening of chemical databases identified 156 compounds with potential antileishmanial activity (Castillo-Garit et al., 2018). Jamal et al. used four types of ML methods (NB, RF, J48, and SMO) to model binary data for *L. mexicana* (Jamal and Scaria, 2013). They employed specific accuracy measures for highly unbalanced data sets (many more inactive compounds than active) that quantified an accuracy of around 80% for all algorithms, although it was not clear whether this was for the training or test sets.

While this paper was being prepared, Herrera-Acevedo and coworkers published a ligand and structure-based modelling study of structure-activity relationships in potential drugs for *L. donovani* (Herrera-Acevedo et al., 2020). They used RF to generate binary classification models for 3,159 compounds with antiamastigote activities and 1,569 compounds with antipromastigote activities. The RF models had accuracies of around 75% (50% accuracy corresponds to chance classification).

#### Malaria

SVMs have been used to identify novel 20S proteasome inhibitors as potential drugs against *Plasmodium falciparum* (Subramaniam et al., 2011). Their SVM model was trained on 170 molecular descriptors for 272 inhibitors and noninhibitors of 20S proteasome. A nonlinear radial basis function SVM kernel provided better classification accuracy than a linear kernel. Fivefold cross-validation accuracy was 97% and subsequent molecular docking was used to refine the short list of inhibitors.

A subsequent study by Jamal and coworkers used ML methods to model high-throughput screening data on apicoplast inhibitors of malaria (Jamal et al., 2013). They employed NB, RF, and DT methods to predict the antimalarial properties of 22,335 active and 197,373 inactive compounds (large data set imbalance). They used the balanced classification rate (BCR, the average of sensitivity and specificity) metric that is applicable to unbalanced data to compare the three ML methods. Given that the models were trained on the same data and descriptors, the three methods generated similar prediction accuracy of 60%, only marginally higher than chance (50%).

An ML study of antimalarial compounds with an unusual mode of action was reported by Maindola et al. Erythrocyte invasion by *Plasmodium*, mediated by the interaction between the apical membrane antigen (AMA1) and rhoptry neck RON2L) proteins, is essential for the parasite to invade host cells (Maindola et al., 2015). Data were obtained from a qHTS bioassay based on AlphaScreen technology that used a recombinant His-tagged AMA1 and a biotinylated RON2L peptide to assess disruption of this molecular interaction. They trained several supervised classifiers, NB, SVM, DT, and RF, using these data. The training set consisted of 588 active and 284 968 inactive molecules and the test set 147 active and 71,241 inactive molecules, respectively (another very unbalanced binary data set). When scored using G-means, a metric suitable for highly unbalanced data sets, the three traditional ML methods performed better (0.8 ± 0.02) than NB (0.73). They used the model to screen a traditional Chinese medicine database and identified 216 potential inhibitors of the AMA1-RON2 interaction. Some hits were already known to be antimalarials and the model predicted that their mode of action is inhibition of the AMA1-RON2 interaction.

An unusual evolutionary SVM method was used to model 17 antimalarial binary data sets, with imbalances (ratio of inactives to actives) of between 3 and 5, with accuracies between 70 and 95% (Viira et al., 2016). The models were used to screen a larger database of candidate molecules and the *in silico* model predicted 27 compounds to be active, 17 of which were confirmed experimentally.

Bharti et al. also reported ML models of antimalarial activity (Bharti and Lynn, 2017). Their dataset consists of 305,803 compounds including 18,126 biologically active compounds against apicoplast formation in *P. falciparum* (again a very unbalanced binary data set). They used a feature selection method to reduce the dimensionality of GLM, kNN, SVM, RF, and DT models and suitable accuracy metrics for unbalanced data sets (F1-score and MCC). The kNN and GLM methods were the least accurate at predicting the test of molecules not used to train the models.

Lawrenson and colleagues used simple, linear MLR and PLS methods to model a set of 44 novel 4-aminoquinoline compounds from a patent that showed activity against a multidrug-resistant (MDR), chloroquine-resistant, and sensitive strain of *P. falciparum* (Lawrenson et al., 2018). Although the dataset was small and not particularly diverse, the linear regression models were effective at predicting the antimalarial properties of the test set, with *r* ^2^ values >0.8 for both strains.

Sahu et al. used the KPLS ML method to model activities of compounds active against *P. falciparum* (Sahu et al., 2020). They trained the model on 57 thiazolyl triazine derivatives and molecular fingerprint descriptors. Using four latent variables in the model, they could predict a 10-compound test set with an *r* ^2^ value of 0.79 and RMSE of 0.33 log activity, although the test set data were poorly distributed. As with de Sousa et al. above, they also mapped the contributions of molecular features to biological activity.

Lima et al. used shape-based and ML methods to model antimalarial compounds (Lima et al., 2019). These models were used to virtually screen a large commercial database of drug-like molecules and identified 10 promising hits that were validated experimentally against asexual blood stages of both sensitive and MDR *P. falciparum* strains. Three compounds showed potent antiplasmodial activity, with EC_50_ ≤ 700 nM, selectivity indices >15, and good *in vitro* inhibition of *P. berghei* ookinete formation.

#### Tuberculosis

Periwal and colleagues were among the first to report models of drug efficacy using traditional ML methods (NB, RF, SVM, and DT) trained on physicochemical properties of compounds from three publicly available bioassay screens of *Mycobacterium tuberculosis* inhibitors (Periwal et al., 2011). The data sets were converted into binary active/inactive sets that were highly unbalanced (many more inactives than actives). Using accuracy metrics suitable for unbalanced data sets (Matthews correlation coefficient and BCR) they found that the RF algorithm provided higher performance than the other ML methods.

Multiple linear regression modelling and single ANNs, ensembles of ANNs, and associative neural networks (AsNNs) have been compared in their abilities to model four different data sets of compounds active against *M. tuberculosis* (Ventura et al., 2013). Unlike most of the other ML studies reviewed here, these were continuous regression rather than binary classification models. The comparison between modelling methods largely disclosed a degree of nonlinearity in the structure-activity relationships. The initial data set of 173 compounds was also subdivided into smaller sets based on the chemotypes and were represented by 96 calculated descriptors. The data sets were divided into training and internal test sets consisting 20–50% of the data set. Models were additionally validated by predicting the activities of an external data set of 22 hydrazide derivatives compounds from the Novartis TB public access database. Based on SE and RMSE values (Alexander et al., 2015), they found that MLR models and ANNs had similar accuracies in predicting the internal test sets, and ensemble ANNs and AsNNs had slightly better accuracies. All methods had similar accuracies in predicting the activities of the 22-compound external test set. Hassan and Khan reported classification models for antitubercular activity using similar ML methods trained on a dataset of 312 active and 300891 inactive molecules and 179 molecular descriptors (Hassan and Khan, 2017). The RF and DT models had the best performance (88–92%) based on the BCR metric.

Traditional ML methods, AsNN, RF, kNN, and XGBoost, were also used to generate models predicting antitubercular activities using a training set of 6,337 compounds (Kovalishyn et al., 2018). These molecules were derivatives of azoles, isoniazids, indoles, and others that exhibited MIC values from 1.5 nM to 100 μM. Unusually, both regression and classification models were generated. The binary classification training set contained 2,705 high activity and 3,632 low activity molecules using an MIC = 10 μM decision boundary. For classification, all ML methods generated models with similar test set prediction accuracy of 80 ± 2%. The AsNN and XGB regression models trained on a smaller 510 data set that was split into training (408) and test (102) sets had similar prediction accuracies for the test set, with *r* ^2^ values of 0.70–0.73 and RMSE values 0.51–0.54 log activity. The consensus classification model was used to screen a database of 165 isoniazid derivatives with different substitution patterns, yielding 18 compounds with predicted µM activities. Seven of these were active against a wild type TB strain and three were active against a strain resistant to isoniazid and rifampicin. Subsequent studies of isonicotinic acid hydrazide derivatives generated models that could predict the test set with balanced accuracies of 67–79% within the domain of applicability of the models (the region of chemical space for which the model is most accurate) (Kovalishyn et al., 2020).

An unusual application of ML to discover antitubercular peptides, using peptide sequence features, has been reported recently (Usmani et al., 2018). They trained SVM, RF, DT, and NB models on different amino acid sequences. The resulting ensemble classifiers achieved an accuracy of 73% and 0.80 AROC for the main dataset of 246 peptides (length 5–61 amino acids) that were active against *Mycobacterium* and provided similar accuracies for a secondary dataset. Again, the RF model had significantly higher prediction accuracy for a validation set than models generated by the other ML methods. They also developed a webserver (http://webs.iiitd.edu.in/raghava/antitbpred/) that allows users to predict peptide antitubercular activity.

In a subsequent study (Khatun et al., 2019), the same data set was used to model the structure-activity relationship using a different representation of the peptide sequences, two-sample-logo representations generated by a web server (http://www.twosamplelogo.org/). In this approach, amino acid preference at each position is denoted by a symbol, where large symbols identify common or conserved residues. Models of peptide activity generated by SVM and RF showed good predictivity for a test set, with accuracies between 77 and 80% for the largest data set. The peptide sequence logos were analyzed to identify the most beneficial peptide features. A related study of anti-TB peptides was reported by Manavalan et al. (2019). Here they used sequence and physicochemical features to encode the peptides and compared the predictive power of a new type of RF method, extreme random trees (ERT), with traditional ML methods, GB, k-NN, LR, RF, and SVM. The binary activity data were balanced and, across nine types of feature encoding, the kNN and LR models had the lowest accuracies and the RF, ERT, and GB models the best (MCC 0.6–0.7 and accuracies of 80–91%)).

In a recent study, Pires and Ascher used mycoCSM, a graph-based signature approach, to rapidly identify compounds likely to be active against mycobacteria (Pires and Ascher, 2020). They trained their ML models on experimental MIC values for over 15,000 compounds across eight mycobacterial species. DT models of antitubercular activity could recapitulate the properties of compounds in the test sets very well. Across the eight species, *r* ^2^ values varied between 0.58 and 0.77 and RMSE values between 0.30 and 0.61 log activity units. Their models have been made accessible by a web server (http://biosig.unimelb.edu.au/myco_csm) that allows users to submit molecules for quick prioritization and screening.

#### Multiple Tropical Disease Studies

Singh et al. reported the use of a suite of relatively novel classifiers to identify inhibitors of trypanosomal N-myristoyltransferase (NMT) (Singh et al., 2016). They compared the performance of the IB1 classifier (a type of nearest neighbor algorithm), Nonnested Generalized Exemplar (assigns generalized exemplars without nesting), Best First Tree algorithm (a modification of standard DT), and logistic regression. The active dataset (120 molecules) consisted of reported inhibitors of trypanosomal, leishmanial, and plasmodial NMT. The inactive dataset (6,160 compounds) consisted of decoys generated for each active molecule. Unfortunately, the authors did not account for the highly unbalanced nature of the data set by using suitable scoring metrics. The >99% accuracy measures reported were therefore unrealistic, as this could be achieved by simply assigning all compounds to the inactive class. A more realistic G-means metric (geometric mean of sensitivity and selectivity) generates accuracies for models from all 4 ML methods of ∼88% for the test set. The 54,275 molecules in Maybridge small molecule library were screened for potential trypanosomal NMT inhibitors, although no check was made as to whether these were within the domain of applicability of the model. However, subsequent 3D QSAR modelling, docking to the enzyme structure, and molecular dynamics simulation identified several new leads with low micromolar *in vitro* activity against *L. donovani* and *T. brucei.*

### Bayesian Models, Clustering, and Visualization

Bayesian methods allow prior knowledge on the properties of molecules to be used to generate models predicting biological activity. When combined with sparsity inducing priors, they are also able to optimize model complexity and reduce the number of adjustable parameters in models, improving interpretability and ability to generalize to new data (Burden and Winkler, 1999; Winkler, 2004; Burden and Winkler, 2008; Winkler, 2018). Models that employ Bayesian methods have been employed surprisingly frequently to design or discover new drugs for NTDs. This may be due in part to the dominance of Ekins and his collaborators in this research space, who use Bayesian methods extensively.

#### Malaria

Wicht et al. applied Bayesian classifiers to model HTS data for beta-hematin inhibition and *in vitro* antimalarial (*P. falciparum*) activity (Wicht et al., 2015). By using different cut-offs that defined the active compound class, that changed the balance of the training and test set, they could achieve 70–94% correct prediction of the tests set. They also validated their optimized Bayesian model by screening a database of 1510 FDA approved drugs that largely occupied similar chemical space to that of the training set. The model placed all six clinical antimalarials, plus quinidine barbiturate and hydroxychloroquine in the top 2.1% of the 1,510 compounds.

#### Tuberculosis

Periwal et al. were among the first to compare NB methods to three other ML methods trained on physicochemical properties of compounds from three publicly available bioassay data sets for *M. tuberculosis* (Periwal et al., 2011). They extended this research to the case of extremely unbalanced tuberculosis data sets (Periwal et al., 2012). As with many other studies reported her, in both cases the assay data were converted to binary classes using a suitable activity cut-off value. They overcame the class imbalance by using a wrapper class to convert the existing algorithm into cost sensitive one and the BCR measure to provide balanced accuracy estimates for unbalanced datasets.

Subsequently Yu and Wild compared the performance of a collection of methods, associative classification mining (ACM), which are popular in the data mining community, with NB and SVM (Yu and Wild, 2012). They modelled a data set of 3,779 anti-TB compounds that were classified into active and inactive groups using a minimum inhibitory concentration (MIC) <5 μM. Classification was based on predictive association rules (CPAR), multiple association rules (CMAR), and association rules (CBA). Based on the F1 score that is appropriate for unbalanced data sets, the ACM methods produced similar results to those of SVM and NB.

Tiwari and coworkers used NB to model HTS data for inhibitors of fructose bisphosphate aldolase, an enzyme essential for the glycolysis pathway in *M. tuberculosis* (Tiwari et al., 2016). Kumari et al. used genetic algorithms with correlation-based feature selection to derive predictive models of serine protease inhibitors of TB using NB and other machine learning algorithms (Kumari et al., 2020). The model was used to screen a library of 918 phytochemical compounds and identified 126 potential antitubercular agents.

**FIGURE 2:**
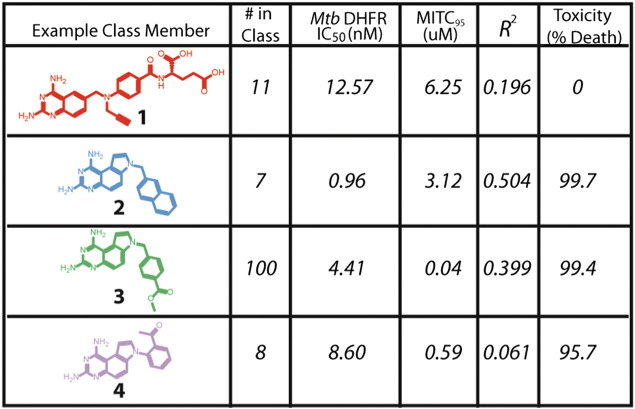
Novel inhibitors of DHFR with *in vitro* efficacy against *M. tuberculosis.* Adapted with permission from Santa Maria et al. (2017). Copyright (2017) American Chemical Society.

Santa Maria and coworkers reported a novel node of action and lead discovery paradigm that used HT biophysical profiling against a broad range of targets and machine learning to identify molecular features for targets for a given phenotypic screen (Santa Maria et al., 2017). They used NB modelling, as this ML method is less sensitive to noise and false negative rates than alternatives. They applied the method to screen 55,000 compounds in 24 internal antibacterial phenotypic screens and against 636 bacterial targets. The ML models identified relationships between phenotype, target, and chemotype for known antimicrobial agents. Specifically, they identified novel inhibitors of dihydrofolate reductase that exhibited nM efficacy (Figure 2) against *M. tuberculosis*.

#### HIV

As HIV is a global problem, there has been intense activity directed towards finding drugs to control this disease. It is beyond the scope of this review to summarize this work; however comprehensive reviews of the application of computational modelling, QSAR, and ML to HIV drug discovery have been published (De Clercq, 2007; Gu et al., 2014; Kumari et al., 2017). To exemplify one approach, Ekins and their collaborators applied their NB methods to a dataset of compounds inhibiting HIV (Zorn et al., 2019). They trained Bernoulli Naïve Bayes, traditional ML, and DL models using both cell-based and target-based (RT DNA polymerase) assay data that were converted into binary (active/inactive) sets using an appropriate cut-off concentration. The cell-based and target-based assay results were correlated (*r* = 0.44) and the data sets used to train the models. Based on balance insensitive metrics, all methods were equally successful in predicting the properties of the test set, supporting the observation that the descriptors have a much larger impact on model quality than the modelling algorithm (Winkler and Le, 2017).

#### Multiple Tropical Disease Studies

Since 2013, Ekins and colleagues have published a relatively large number of NB, RP, and SVM models of molecules acting against various NTDs, using the same datasets to demonstrate the effects of different ML algorithms, data set sizes (Ekins et al., 2014b), and complexities (Ekins et al., 2014c; Lane et al., 2018). They used data fusion to analyze large sets of compounds active against TB (Ekins et al., 2013a; Ekins et al., 2013b). They employed Laplacian-corrected (sparsity inducing) Bayesian classifiers, SVM, and RP to model a training set of 2,273 compounds and validated the predictive power with two external test sets of sizes 1,924 and 1,777 molecules (the latter all active). The Bayesian model could predict a higher number of active compounds from the 1,777 active compounds’ test set than the other ML methods. They further combined antitubercular activity and cytotoxicity (to Vero, THP-1, and HepG2 cell lines) to generate Bayesian models that identified drugs active against TB that were also less cytotoxic (Ekins et al., 2014a). They again used a set of 1,924 commercially available molecules, evaluated for antitubercular activity and cytotoxicity, with hit rates of 3–4%. They demonstrated that models incorporating antitubercular and cytotoxicity data significantly enrich selection of nontoxic actives.

More recently, Ekins and coworkers deployed their Bayesian ML methods to model and predict the activity of small molecules against trypanosomes and Ebolavirus (Ekins et al., 2015a; Ekins et al., 2015b). To discover antitrypanosome drug leads, they trained a Bayesian model on literature compound data and a subset of the Broad Chagas dose response data set, using the EC_50_ values <1 μM to define actives (Ekins et al., 2015a). This binary training set contained similar numbers of active and inactive compounds (well balanced) and 5-fold cross-validation showed the model had a prediction accuracy >80%. The model was used to screen 7,200 molecules from eight small chemical libraries, 97 of which were tested and 11 found to have EC_50_ < 10 μM. The five most active molecules, verapamil, pyronaridine, furazolidone, tetrandrine, and nitrofural, had *in vitro* EC_50_ values < 1 μM and also showed good activity *in vivo* in a *T. cruzi* mouse model.

For Ebola virus, these researchers used a broadly similar Bayesian approach to model 868 molecules from the viral pseudotype entry assay and the Ebola replication assay (Ekins et al., 2015b). The binary data set was very unbalanced, with only 4% of the compounds in the active class. Although the model could predict activity with >80% accuracy using 5-fold cross-validation, an accuracy metric more appropriate for unbalanced data sets (BCR, MCC or G-means) would have given a more realistic estimate of the model performance. Nonetheless, the model identified three compounds, quinacrine, pyronaridine, and tilorone, subsequently tested *in vitro* with EC_50_ values of 350, 420, and 230 nM. Subsequent work identified the antimalarial drug arterolane (IC_50_ = 4.53 µM) and the anticancer clinical candidate lucanthone (IC_50_ = 3.27 µM) as novel drug leads that have Ebola inhibitory activity in HeLa cells and low cytotoxicity (Anantpadma et al., 2019).

Korotcov and coworkers reported a comprehensive ML study across a range of biological assays that included bubonic plague (*Yersina pestis)*, Chagas disease (*T. cruzi*), TB (*M. tuberculosis*), and malaria (*P. falciparum*). The assay data were converted into binary form (active/inactive) (Korotcov et al., 2017). The datasets varied greatly in balance, with plague and malaria having very low percentages of active compounds. They compared the performance of Bernoulli NB (BNB) methods with traditional ML methods and DNN when trained using molecular fingerprint descriptors. Using suitable metrics for unbalanced data (F1-score and Matthews correlation coefficient) they found that BNB was as effective at predicting the properties of the test set compounds as the other ML and DNN methods.

While NB usually shows performance similar to that of other ML methods such as SVM and RF, incorporation of Bayesian methods in other ML algorithms can have substantial benefits in terms of optimizing model complexity to avoid bias (underfitting) or variance (overfitting) problems. When sparsity inducing (Laplacian) priors are used with these methods (e.g., Bayesian regularized neural networks or relevance vector machines), models have fewer parameters and neural networks have fewer effective weights, thus improving predictions and interpretability of the model and descriptors (Burden and Winkler, 1999; Burden and Winkler, 2008; Burden and Winkler, 2015).

The above studies have shown how accessible ML methods trained on public domain data sets of activity for diverse NTDs can be used to provide some mechanistic or structure-activity information and also screen much larger collections of organic molecules for potential lead. They provide very useful proof of concept but do suffer from some shortcomings. They did not explore a wider range of ML methods and descriptor classes to exploit advantages of some of the newer techniques. They also mostly convert continuous data into binary (active/inactive) data sets and so lose information intrinsic to the data. They also screen large databases without specifying whether the members lie in or close to the domain of applicability of the models in most cases.

### Deep Learning Models

Deep learning algorithms excel at analyzing image-based data to extract subtle features. They are being adopted rapidly for disease diagnosis and analysis of data from x-ray, CT, and MRI imaging. There are an increasing number of papers now employing deep learning methods for diagnosis of NTDs (e.g., Gao and Qian, 2018; Ting et al., 2018; Khalighifar et al., 2019; Rajaraman et al., 2019; Yang et al., 2019; Fuhad et al., 2020) that are beyond the scope of this review. Although deep learning methods offer state-of-the-art performance in modelling the biological properties of drug-like data for next generation drugs (Ferreira and Andricopulo, 2019; Lavecchia, 2019), they have not yet been widely adopted by the NTD research community.

One of the first studies that employed deep learning methods was reported by Korotcov et al. (2017). They compared the performance of several classic ML methods, NB, LLR, DT, RF, and SVM, with deep neural networks (DNN). They modelled several data diverse sets including aqueous solubility, bubonic plague, Chagas, tuberculosis, and malaria using molecular fingerprints (FCFP6) as descriptors, which had been modelled previously by Clark et al. using traditional and Bayesian ML methods (Clark et al., 2015). They assessed whether DNN methods had advantages over the other ML algorithms using traditional metrics. This study focused on comparing methods useful for discovery of NTDs and they did not actually apply the models to discovery. Although a very useful proof of concept, the study had several shortcomings. The biological data were converted in active/inactive classes using a threshold value that varied for each data set. Most of the resulting data sets were moderately to severely unbalanced. Korotcov et al. concluded that DNNs had higher predictive performance than the next best algorithm, SVM. However, using the accuracy metrics for unbalanced binary data sets (F1-score and MCC) shows that, for almost all data sets, only NB and DT models had significantly lower prediction accuracy for the test set. This is consistent with descriptors having the largest impact on model quality, with different ML algorithms giving models with very similar performance if trained using the same descriptors (Winkler and Le, 2017). An interesting follow-up study would be to use the ML and deep learning methods to generate regression models of the biological end points and use the models to screen a library of candidates to discover new potential drugs.

A subsequent study by Lane and colleagues compared the abilities of the same traditional, Bayesian ML and deep neural networks to model and predict the efficacies of tuberculosis drug candidates (Lane et al., 2018). Again, the quantitative data were converted to binary active/inactive data using three different cut-off concentrations. Five types of molecular fingerprint were also used to train the models, ECFP6 and FCFP6 fingerprints, MACCS keys, and RDKit and Toxprint descriptors. However, no sparse feature selection was performed to reduce the dimensionality of the models. The training and test sets were again highly or moderately unbalanced and, not surprisingly, the authors reported that the AROC values and other precision and accuracy metrics did not necessarily correlate with the F1-score and MCC metrics. Bayesian machine learning models trained on literature TB data generated by different laboratories (18,886 compounds in training set) performed on average as well as deep neural networks in predicting the activities of molecules in the external test sets. There were sometimes large differences in test set predication accuracies within the DNN models and between DNN and other ML, depending on the activity cut-off and descriptors used. Lane et al. proposed that these machine learning models could help prioritize compounds for testing *in vitro* and *in vivo* against tuberculosis.

Ekins et al. also used their suite of ML methods, which included DNNs, to model HIV drug activity data from the NIAID ChemDB HIV, Opportunistic Infection, and Tuberculosis Therapeutics Database (Zorn et al., 2019). They modelled HIV-1 wild type cell-based and reverse transcriptase DNA polymerase inhibition assays that were moderately correlated. Again, they compared predictive abilities of multiple machine learning approaches and demonstrated that SVM, deep learning, and a consensus of all models gave comparable predictions accuracies, as assessed by 5-fold cross-validation and test sets. This study is again consistent with previous studies of training and testing with multiple data sets that show little difference between support vector machine and deep neural networks models trained on the same data and descriptors.

A new AI system, DeepMalaria, for discovery of antiplasmodial drugs, has recently been reported by Keshavarzi Arshadi and coworkers (Keshavarzi Arshadi et al., 2020). A graph-based model was trained on 13,446 antiplasmodial hit compounds from GlaxoSmithKline dataset. The model was validated by predicting hit compounds from another compound library and an approved drug repurposing library. DeepMalaria identified all compounds with nanomolar activity and 87.5% of the compounds with greater than 50% inhibition. One hit compound inhibited all asexual stages of *P. falciparum*, making it a strong candidate for further optimization.

Finally, a very recent and successful use of deep neural networks was reported by Stokes and coworkers in Cell (Stokes et al., 2020). In a study aimed at discovering new antibiotics, they trained a deep neural network to identify molecules with relatively broad-spectrum antibacterial activity. They applied a model to multiple chemical libraries and found an existing drug, halicin (**1**), 
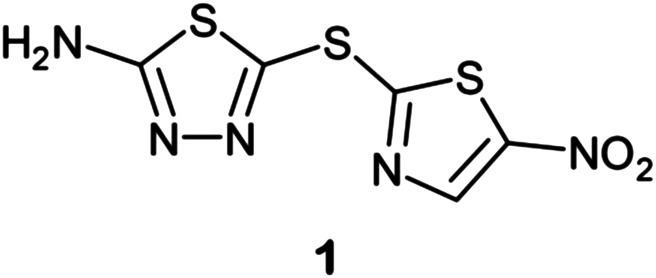
which was structurally distinct from conventional antibiotics and displays wide bactericidal activity *in vitro* and *in vivo*. Halicin operated by a novel mechanism of action, dissipation of the transmembrane ΔpH potential in bacteria, and was also very effective against *M. tuberculosis*. They also screened 107 million compounds from the ZINC15 database and identified eight antibacterial compounds that were structurally dissimilar to known antibiotics. Two of these, ZINC000100032716 and ZINC000225434673, displayed potent broad-spectrum activity and overcame an array of common resistance factors. This was the first successful demonstration of the use of DNN to repurpose existing drugs and discover new drug leads.

### Other AI Methods

Genetic algorithms (GAs) and genetic programming (GP) are very effective at exploring very large feature spaces. They have been used frequently to choose subsets of descriptors for ML models of bioactivities of small molecules. Little genetic algorithm research has appeared yet in the NTD literature. Kumari et al. reported the use of GP approaches to elucidate the role of descriptors in models of serine protease inhibitors as antituberculosis drugs (Kumari et al., 2020). They modelled a library of 918 phytochemical compounds as potential serine protease inhibitors using the RF ML algorithm. Their best RF model trained on descriptors chosen by a GA identified 126 antitubercular agents out of the 918 phytochemical compounds. The genetic programming symbolic classification method they employed optimized descriptors and provided an equation for the mathematical model.

## Perspective

Impressive progress in the application of AI and ML methods to most areas of science, technology, and medicine strongly suggests a much larger role for these methods in the discovery of new treatments for NTDs in the near future. The above illustrative examples show how effective these methods can be in finding new lead compounds. It is also clear from the literature that the rate of adoption of these methods is increasing significantly. This is driven by increasing use of high-throughput screens, increased power and availability of open-source ML methods, and development of novel DL methods that generate descriptors, model relationships, and perform inverse mapping of models to lead compounds. Some of these developments are discussed below.

### Developments in Assay Technologies and Mechanism of Action Studies

Assays drive drug discovery and provide the data sets used to train AI and ML models that leverage these data to design or discover new drug leads. Robust identification and characterization of potential leads require biochemical, biophysical, and cellular data that are increasingly generated by high-throughput methods. Recent efforts have resulted in miniaturized assays arrayed in microtiter plates that can test >100,000 samples/day; microtiter plate-free formats with encoded libraries that can potentially screen billions of compounds; a search for new drug modalities; and emphasis on more disease relevant screens using complex cell models of disease states. Developments in this area were reviewed recently (Busby et al., 2020). Development of HTS assays for NTDs was reported by Qing et al. for dengue fever (Qing et al., 2010). Dengue virus-like particles were constructed using viral structural proteins plus a luciferase reporter. High titer particles (>10^6^ foci-forming units/ml) were obtained whose infection could be blocked by antibodies against viral envelope proteins and by viral NS5 polymerase inhibitors. The infection assay was run in a 384-well format and provided a simple, robust, and rapid response suitable for screening large chemical libraries for compounds inhibiting dengue entry, translation, and replication.

The abilities of machine learning methods to improve information extraction from imaging data are finding new applications in mechanism of action studies. Because these methods do not work well with heterogeneous cellular phenotypes and require human training, Ashdown and coworkers reported a combined human- and machine-labelled approach for data from mixed human malaria parasite cultures (Ashdown et al., 2020). Trained on high-throughput and high-resolution screening data, their approach tolerates natural parasite morphological heterogeneity and correctly orders parasite developmental stages. They successfully detected and clustered drug-induced morphological outliers by mechanism of action, opening the door for faster and more robust cell-based drug discovery.

### Quantitative ML Models and Multitarget/Multidrug Strategies

As this review has illustrated, many AI and ML models of drugs and targets for NTDs have been binary classification models (e.g., active/inactive). It is not clear why continuous data sets have been converted into binary data sets for modelling purposes as this loses considerable information. One reason may be that binary classification model generates relatively high-performance metrics compared to continuous regression models, as the model is essentially fitting only two clusters of data points rather than the whole span of data. Sometimes binary models provide misleading estimates of model predictive power, especially when data sets are unbalanced. There are accuracy measures such as G-means (geometric mean of sensitivity and specificity) and F1-factor (harmonic mean of sensitivity and specificity) that provide more realistic estimates of predictive accuracy that are often not used. Other methods of removing class bias are under sampling of the majority class and imputing new data for the minority class.

Ultimately, using computational methods to generate effective descriptors and to map them to biological activities using continuous data offers significant advantages. These include better estimates of the contributions of specific molecular features to the biological activities and the ability to predict specific EC_50_, LD_50_, and other values across several orders of magnitude. This provides a lot more information about differences between molecules in the active class than do binary classification models. Given the relatively large amount of effective ML modelling software available in the public domain, quantitative modelling is an important ML application for discovery of more effective drugs for NTDs.

The concurrent usage of multiple medications is necessary for some diseases, especially to manage or minimize the development of drug resistance. Resistance to mainstream drugs is a particular problem with NTDs (Pramanik et al., 2019). Drug resistant malaria and TB are common, with almost 500,000 new cases of multidrug-resistant tuberculosis in 2016 and a 45% mortality rate worldwide. ML methods are being used to predict resistance phenotypes (Wheeler et al., 2020) and the effects of multidrug dosing and drug synergies. A novel application of ML to predict synergistic combinations of antimalarial drugs was reported by Mason et al. (Mason et al., 2018) The discovery of synergistic combinations of antimalarial drugs is potentially very important, but an exhaustive experimental screen of every antimalarial drug with all others is not tractable. Mason and coworkers used SVM to model a dataset of 1,540 antimalarial drug combinations, of which 22% were synergistic. Cross-validation showed that synergistic predictions are enriched between 2.7- and 1.5-fold compared to random selection. This depends on whether compounds in a combination are known from other combinations in the training data or are entirely novel combinations.

Siddique et al. reported the use of SVM, GB, and LR to estimate the generalized propensity score, the probability of receiving a specific treatment (Siddique et al., 2019). They modelled data from 9,290-patient multidrug-resistant TB treatment outcomes, from 31 observational studies, to compare the results of the different treatment regimens. The different ML models often agreed on the best regimens but produced sometimes differing estimates of probabilities of treatment success. A novel hybrid approach has been suggested recently by Riches and coworkers (Riches et al., 2020). They described new anti-giardia agents that contain more than one toxophore and are capable of killing pathogens by multiple mechanisms of action.

### Use of ML and DL Methods to Generate More Effective and Interpretable Models and Improve Docking Scores

As the author stated, the quality of models and their predictive power is largely controlled by the relevance and interpretability of the molecular descriptors used to train them. By employing chemically interpretable descriptors that can be effectively mapped back onto prototype molecules, chemists gain substantial insight into how to improve activity and selectivity and reduce toxicity of drug candidates. There is a trend away from using effective but arcane molecular descriptors to train ML models of drug activity because these models often provide little insight into how to improve lead molecules. Molecular fragments and fingerprint methods are being increasingly used to provide both efficiency and interpretability (Muegge and Mukherjee, 2016; Hessler and Baringhaus, 2018; Kleandrova and Speck-Planche, 2020), and kernel-based methods like those reviewed above are also very useful. As alluded to, DL methods are providing paradigm shifts in the generation of effective descriptors and to a lesser extent (at least for the present) in interpreting molecular features in ways that medicinal chemists can use.

ML methods have also been used to improve, augment, or even replace molecular docking methods used to screen chemical libraries for compounds binding to specific protein targets. For example, Kinnings and coworkers reported use of SVMs to improve the docking scores of compounds (Kinnings et al., 2011). They generated models that mapped individual energy terms from molecular docking to the known binding affinity of compounds from HTS experiments. This improved the predicted binding affinities from docking programs. They applied the method to predict the binding energies of inhibitors of *M. tuberculosis* InhA. This identified the potential for phosphodiesterase inhibitors to be repurposed anti-TB drugs. The methods are generally applicable to other NTD target structures. DNNs are being increasingly used to emulate docking of small molecules to proteins. Jastrzębski et al. recently reported the application of a deep neural network to predict docking outputs directly from a two-dimensional compound structure (Jastrzębski et al., 2020). Their procedure is orders of magnitude faster than typical docking software, and it provides interaction fingerprints for ligand–receptor complexes. This development greatly facilitates screening of vast compound libraries, or libraries of existing drugs, clinical trials candidates, and approved natural products that have already been in man, for repurposing in NTDs.

### Broad Screening of Large Chemical Libraries or Leads, Particularly Repurposing Drugs

The repurposing of registered drugs, clinical trials candidates, and approved natural products, whose safety and pharmacokinetics in man are known, is a rational approach to rapid discovery of new drugs for NTDs. Drug repurposing (repositioning) discovers new disease indications for previously approved drugs, especially relevant for NTDs where the estimated US$1-1.3Bn required to get a new drug on the market is more difficult to raise. This discovery paradigm has been dramatically accelerated by the COVID-19 pandemic as has been described recently (Guy et al., 2020). Computational methods of drug repurposing have rapidly gained favor because of their speed, low cost, improved accuracy, and accessibility due to the impressive amount of ML software available in the public domain. The rationale and principles for repurposing drugs for new diseases have been reviewed (Pushpakom et al., 2019). Very recent reviews have summarized HT and computational repurposing for NTDs (Hernandez et al., 2018; Andrade et al., 2019; Bustamante et al., 2019).

A very important but sometimes overlooked fact is that ML models of biological responses have specific domains of applicability. These are determined by the ranges of the molecular descriptors and the range of biological activities used to train models. Larger and more chemically diverse training sets will generate models that have larger domains of applicability and will be able to generalize to new data more broadly than smaller, less diverse training sets. If members of virtual screening libraries do not have properties that lie within, or at least close to, those of the training compounds, then predictions made by the models will be less reliable.

Schuler and colleagues have published a useful *in silico* study of drug repurposing for Ebola virus (Schuler et al., 2017). They also provide a helpful list of software and servers used for computational drug discovery. Ebola virus is a particularly difficult repurposing problem mainly due to the small amount of structure-activity data available in the literature with which to train models. As the studies reviewed in *Case Studies Using AI and ML to Discover New Drugs for NTDs* show, most other NTVs have large or very large databases of compounds with associated biological activity data. These resources can be used to train ML models with broad domains of applicability that can be used for drug repurposing. However, drugs whose molecular features lie outside these domains usually have their biological properties poorly predicted. Schuler et al. stressed the importance of multitargeting approaches, especially when preclinically or clinically validated. Several of the approaches they reviewed are broadly applicable to other pathogens, outbreaks, epidemics, and pandemics and to general drug discovery and development.

### Use of Evolutionary Methods and Other AI Methods to Discover NTD Leads

Although almost all applications of AI methods to discovery of drugs for NTDs have focused on ML methods, there are additional AI technologies that show promise. Wang et al. have summarized the current and future impact of AI methods on infectious diseases (Wong et al., 2019). Given the immensity of drug-like chemical space (∼10^60^ compounds) and the need to optimize several properties simultaneously to generate good NTD drug leads, evolutionary methods are beginning to be employed in mainstream drug discovery. These represent the structural and physicochemical properties of molecules by mathematical “genomes.” They use a combination of desirable (e.g., activity, pharmacokinetics) and undesirable (e.g., toxicity, cost) properties as a fitness function to be optimized. By assessing the fitness of small libraries, mutating the genomes of the best candidates, and synthesizing new pools of improved candidates, drug leads can be rapidly optimized. Depending on the type of mutation operator used, large jumps into novel chemistry space can be achieved (scaffold hops). Although there are no examples of evolutionary optimization of drugs for NTDs yet, the current state of the art for mainstream drug discovery has been reviewed recently (Le and Winkler, 2015). An interesting application of evolutionary methods to assess risk factors for Chagas disease was very recently published byHanley et al. (2020). The data were derived from surveys of 64 risk factors believed to be relevant to infestation of households. The results may inform the design of eco-interventions aimed at slowing the spread of Chagas disease.

Autonomous experimental systems are under development in several laboratories. These aim to create a closed loop system that automatically designs and synthesizes molecules that are fit for purpose. Most systems rely on evolutionary methods to perform the successive cycles of optimization until no further improvement is achieved or an acceptance metric is reached. They consist of robotic synthesis methods (or alternatively, a large pool of available compounds), one or more assays to determine the “fitness” of the molecules, a means of mathematically “mutating” members of the fittest populations, and synthesis of these that is carried out by the synthesis robot (or members chosen from the large pool). A robot scientist “Eve,” an automated system using AI to discover knowledge through cycles of experimentation, aims to make drug discovery faster and more economical (Williams et al., 2015). It performs library screening, hit confirmation, and lead generation using QSAR models. Williams and coworkers used econometric modelling to show that Eve outperforms standard drug screening on an economic basis. It employs as fitness measure assays that can be quickly and cheaply engineered using synthetic biology. Conspicuously, Eve has repositioned drugs against parasites that cause tropical diseases. They used a drug library to identify repurposed drugs against malaria, Chagas, African sleeping sickness, and schistosomiasis. In particular, the antimicrobial compound fumagillin, an angiogenesis inhibitor, investigated as an anticancer drug, inhibits growth of *P. falciparum* strains (including resistant strains) and inhibits parasitemia in a mouse model.

Computationally guided discovery of new drugs for treating NTDs has benefitted from sophisticated methods developed for pharmaceutical drugs used to primarily treat major illnesses in the developed world. Although application of HTS and ML methods to discover new drugs for NTDs has lagged behind that for noninfectious diseases, the stage is set for rapid adoption by scientists working on NTDs, especially those in the developing world. The new technologies foreshadowed in *Perspective* bode well for more rapid and informed discovery and, ultimately, design of more potent, selective, and safe drugs for NTDs in the future.
